# Optimizing Conditions for the Production of Bacterial Extracellular Vesicles of *Vibrio vulnificus* and Analysis of the Inner Small RNA Compositions

**DOI:** 10.4014/jmb.2310.10002

**Published:** 2023-12-05

**Authors:** Jeong Heon Park, Suji Song, Soyee Kim, Minjeong Kim, Kun-Soo Kim

**Affiliations:** Department of Life Sciences, Sogang University, Seoul 04107, Republic of Korea

**Keywords:** *Vibrio vulnificus*, bacterial extracellular vesicle (BEV), outer membrane vesicle (OMV), small RNA (sRNA)

## Abstract

Chemical and physical elements affecting the production of bacterial extracellular vesicles (BEVs) of the human pathogen *Vibrio vulnificus* were quantitatively assessed to optimize the conditions for the BEV production by using the western blot quantification for an outer membrane porin OmpU and by fluorescent dye FM4-64. When cells were cultured at 37°C in an enriched medium (2 × Luria Bertani; 2 × LB) in the presence of EDTA, they produced about 70% more BEVs. BEVs were purified from the cells cultured in the established optimal conditions by the density gradient ultracentrifugation. The dynamic light scattering measurement of the purified BEVs showed that the diameter of them ranged from approximately 25 nm to 161 nm. We hypothesized that there may be some features in nucleotide sequences specific to RNAs packaged in BEVs compared to those in cellular RNA molecules. We compared the nucleotide sequences and abundance of sRNAs between in the cellular fraction and in BEVs through next-generation sequencing (NGS). While no distinct feature was observed in the nucleotide sequences of sRNAs between two groups, the length of sRNA fragments from BEVs were significantly shorter than those in cytoplasm.

## Introduction

Bacterial extracellular vesicles (BEVs) are small membranous vesicles that are released by bacteria. These vesicles can be classified into four different types based on their membrane composition and origin [1]. Cytoplasmic membrane vesicles (CMVs) are produced by gram-positive bacteria and are derived from dying cells. In contrast, gram-negative bacteria produce three different types of BEVs. Outer membrane vesicles (OMVs) are produced by the non-lytic release of the outer membrane, while explosive outer membrane vesicles (EBEVs) are produced through the lytic release of the outer membrane. Outer inner membrane vesicles (OIMVs), on the other hand, are generated through the release of both the inner and outer membranes [2].

During growth, gram-negative bacteria constantly release BEVs, which are enriched with outer-membrane proteins and display specific lipid compositions [3]. Studies have revealed that BEVs may contain periplasmic and cytosolic proteins, DNA, and RNA, and transport virulence factors [4-6]. The amount and content of cargo molecules in BEVs vary depending on growth conditions [7]. The production of vesicles depends not only on the genetic background of the producing cells but also on various growth conditions. Environmental factors such as media composition, growth phase, temperature, nutrient availability, oxidation, quorum sensing, exposure to antibiotics, and genotoxic stress can affect the rate of vesicle formation [7, 8].

BEVs can contribute to bacterial survival by breaking down and sequestering antimicrobial molecules [2]. BEVs protect cells from host antimicrobial peptides and phage infection by acting as decoys for their binding [9]. Additionally, BEVs can cause bacterial aggregation and biofilm [10]. Furthermore, BEVs contain protective enzymes such as active β-lactamase which degrades β-lactam-based antibiotics [11] and release nutrients from large complex molecules using various metabolic enzymes, including proteases, glycosidases, phosphatases, and lipases [12, 13].

The presence of BEVs in the surrounding environment is involved in intercellular communication [14, 15]. The small RNAs (sRNAs) found in BEVs have been suggested as potential molecules for interspecies communication, as they have been shown to regulate gene expression in various cell types and species. One key function of BEVs is not only to transport cargo but also to safeguard sRNAs from extracellular RNases and facilitate their delivery to host cells [4]. It was assumed that the small non-coding RNAs (sRNAs) produced by pathogenic *Vibrio* species, including *V. vulnificus*, would have many unknown biological roles in intercellular interactions.

Little has been know about the natures of BEVs in the human pathogen *V. vulnificus* and factors affecting their generation. In this study, as a foundational research, we investigated the effects of various factors on the production of BEVs to obtain the optimal conditions to yield the maximal production from *V. vulnificus*. Additionally, we characterized sRNAs incorporated into BEVs using the next generation sequencing (NGS).

## Materials and Methods

### Bacterial Strains and Growth Conditions

*V. vulnificus* MO6-24/O obtained from pathogenic clinical isolate [16] was used in this study. Cells were grown in the Luria-Bertani (LB) medium at 30°C. To test different environmental factors, *V. vulnificus* cells were also cultured at 25°C or 37°C. Cells were cultured in a regular LB medium or enriched 2 × LB medium (2% Tryptone, 1% yeast extract, 1% NaCl), or in low or high salt LB medium (0.1, 0.2, 0.5, 0.9, or 3.5% NaCl). Polymyxin B was added at the final concentration of 20 μg/ml, and EDTA at 100 μM to the medium. Cyclo-phenylalanine-proline (cFP) was added at the final concentration of 5 mM, FeSO_4_ at 25 μM, 2,2'-dipyridyl at 100 μM, and H_2_O_2_ at 20, 100, 500, or 1000 μM at the OD_600_ value of approximately 0.3. For dynamic light scattering (DLS) and RNA extraction, *V. vulnificus* overnight cultures were inoculated into 1 L of 2 × LB medium (2% Tryptone, 1% yeast extract, 1%NaCl) with 100 μM EDTA in 2 L flasks with an initial OD_600_ of 0.01.

### Trichloroacetic Acid (TCA) Precipitation of BEVs

After inoculation, 1 ml of culture medium was collected every 1 h. The collected culture medium was then centrifuged at 7,000 rpm for 5 min at 4°C and supernatant was filtered with a syringe filter (pore size 0.45 μm, HYUNDAI Micro, Republic of Korea) to obtain bacterial cell free supernatants. Hundred μl of 100% (w/v) trichloroacetic acid (TCA) were added to 900 μl of filtered supernatant and vortexed. After incubating for 30 min at 4°C, the culture was centrifuged at 13,000 rpm for 30 min at 4°C. Taking care not to disrupt the pellet, supernatant was removed, and pellets were washed with 1 ml of 80% cold acetone and centrifuged at 13,000 rpm for 30 min at 4°C. Supernatant was removed and pellets were dried to obtain BEV samples.

### SDS-PAGE and Immunoblot Analysis

Expression of OmpU, which is the porin protein in the outer membrane, served as a marker for BEV containing the outer membrane portion. After TCA precipitation, dried pellet was suspended in 30 μl of phosphate-buffered saline (PBS) and 10 μl of 4× Laemmli’s sample buffer and boiled for 5 min. Then the preparation was subjected to 12% SDS polyacrylamide gel and transferred to a Amersham Protran 0.2 μm nitrocellulose membrane (GE Healthcare UK Ltd., UK). Membranes were blocked with 3% skim milk in Tris-buffered saline with Tween 20 (TBST; 150 mM NaCl, 50 mM Tris-HCl, and 0.1% Tween 20) for 30 min before being incubated overnight at 4ºC with rabbit anti-OmpU antibody [17] (1:1,000 in blocking solution). After incubation with the primary antibody, membranes were washed three times in TBST. Subsequently, goat anti-rabbit IgG-AP (GeneTex, USA) (1:1,000 in blocking solution) was treated as a secondary antibody for 30 min. Then, membranes were washed four times in TBST. Expression of OmpU was visualized using the NBT/BCIP color development substrate (Promega, USA).

### FM4-64 Staining of BEVs

The amount of BEVs were measured by using the lipophilic fluorescent dye FM4-64 (Thermo Fisher, USA) as described previously [18]. For each sample, 90 μl of the filtrate containing BEVs were transferred to the 96-well dark plate. FM4-64 stock solution (1 μg/μl in DMSO) diluted with phosphate-buffered saline (PBS: 137 mM NaCl, 2.7 mM KCl, 10 mM Na_2_HPO_4_, 2 mM KH_2_PO_4_, and pH 7.4) was added to each well at a final concentration of 5 μg/ml and incubated for 10 min at room temperature. BEVs alone and the FM4-64 probe alone were used as negative controls. After excitation at 515 nm, emission at 640 nm was measured with the EnSpire Multimode Plate Reader (PerkinElmer, USA).

### Isolation of BEVs by a Density-Gradient Ultracentrifugation

For BEV isolation from *V. vulnificus* wild-type cells by density-gradient ultracentrifugation, cells were cultivated in 1 L of 2 × LB medium (2% Tryptone, 1% yeast extract, 1% NaCl) with 100 μM EDTA in 2 L flasks with an initial OD_600_ of 0.01. After 8 h of culture, the bacterial suspension was centrifuged at 7,000 ×*g* for 15 min at 4°C. The supernatant was filtered with 0.45-μm-pore size filters (Cat No. 7141 104, Whatman, UK) using Filter Funnel (PALL, USA) and aspirator (EYELA, Japan) to remove residual cells. The cell-free filtrates were concentrated by super absorbent polymer (SAP) (DM100, LG Chem Ltd., Reoublic of Korea) as described previous studies with minor modifications [19]. Briefly, cell-free filtrates were treated with 60 g/l of SAP and incubated on an orbital shaker overnight at 4°C. Remaining supernatants were collected, and concentrated again with SAP. Concentrated cell-free filtrates were filtered with a pore size 0.45 μm syringe filter. The collected BEVs-enriched liquid was transferred to ultracentrifuge tubes (C14277, Beckman, USA) and ultracentrifuged using a ultracentrifuge (Beckman, Optima XE-90) with a SW 41 Ti rotor. Remaining pellet was suspended in 500 μl of HEPES buffer (50 mM HEPES, 150 mM NaCl and pH 6.8).

The overall gradient ultracentrifuge process is schematically depicted in Fig. S1. To prepare density-gradient centrifugation, 1.5 ml of OptiPrep, Germany was placed below the concentrated cell-free filtrates to make a liquid cushion [20]. The preparation was then ultracentrifuged for 3 h at 150,000 ×*g* at 4°C. The supernatant was carefully discarded, leaving 500 μl solution along with the 1.5 ml cushion solution. The remaining 2 ml solution were mixed and transferred to a new ultracentrifuge tube. Optiprep 60%(w/v) iodixanol was diluted with HEPES buffer to create each of 2 ml of 40%, 35%, 30%, and 25%, and 1 ml of 20%. They were over-layered sequentially above the first layer. The prepared gradients were ultracentrifuged for 16 h at 100,000 ×*g* at 4°C. After centrifugation, 1 ml fractions of the density gradient layers were sequentially collected from the top [4].

### Dynamic Light Scattering (DLS) Analysis

DLS measurements were conducted using Zetasizer (Malvern, ZEN3600, UK) in the back-scattering mode of 173° and a temperature of 20°C. The refractive index and viscosity of various Optiprep concentrations were calculated [21, 22]. Each sample was measured three times in 100 μl of sample. Data analysis was performed with Zetasizer software version 7.11.

### Small RNA Extraction and cDNA Library Preparation

RNA extraction was conducted using the miRNeasy mini kit (QIAGEN, 217004, Germany), following the method outlined in the manufacturer's manual titled "Appendix A: Preparation of miRNA-Enriched Fractions Separate from Larger RNAs (>200 nt)". RNA extracts were used to synthesize cDNA libraries with SMARTer smRNA-Seq Kit for Illumina (Cat. Nos. 635029, 635030, 635031, Takara, Japan) according to the manufacturer’s instruction.

### Next Generation Sequencing (NGS) Analysis of Small RNAs

Illumina sequencing was performed by Macrogen (Republic of Korea) with TruSeq. NGS raw data analysis was conducted on the Galaxy platform (22.05) in the Ubuntu 22.04.2 LTS (GNU/Linux 5.15.90.1-microsoft-standard-WSL2 x86_64) environment. To create a workflow through the Galaxy platform installed on a local PC, several programs were required. To check the quality of the data, the FastQC program (0.73+galaxy0) was used. Trimming and filtering raw data by adapter sequence was done using the Cutadapt (4.0+galaxy1) program, and the Bowtie2 (2.5.0+galaxy0) was used to map the sequences in the genome.

To obtain the position with read counts above at least 10 nucleotides and to obtain the gene information of the position, the Python code was written and utilized (Fig. S2).

## Results

### Effect of Physical and Chemical Conditions on the Generation of BEVs

We assessed the effects of various physical and chemical conditions on the generation of BEVs in *V. vulnificus*. At different growth time points, BEVs were purified by the TCA-precipitation method as described in Materials and Methods section. We semi-quantitatively measured the amounts of BEVs using antibody against OmpU, an outer membrane protein that served as a marker of BEVs. We also assessed the amount of BEVs for each condition when the cell culture reached the stationary phase using the FM4-64 staining method as described in Materials and Methods section.

### a. Temperature

**Fig. 1:**
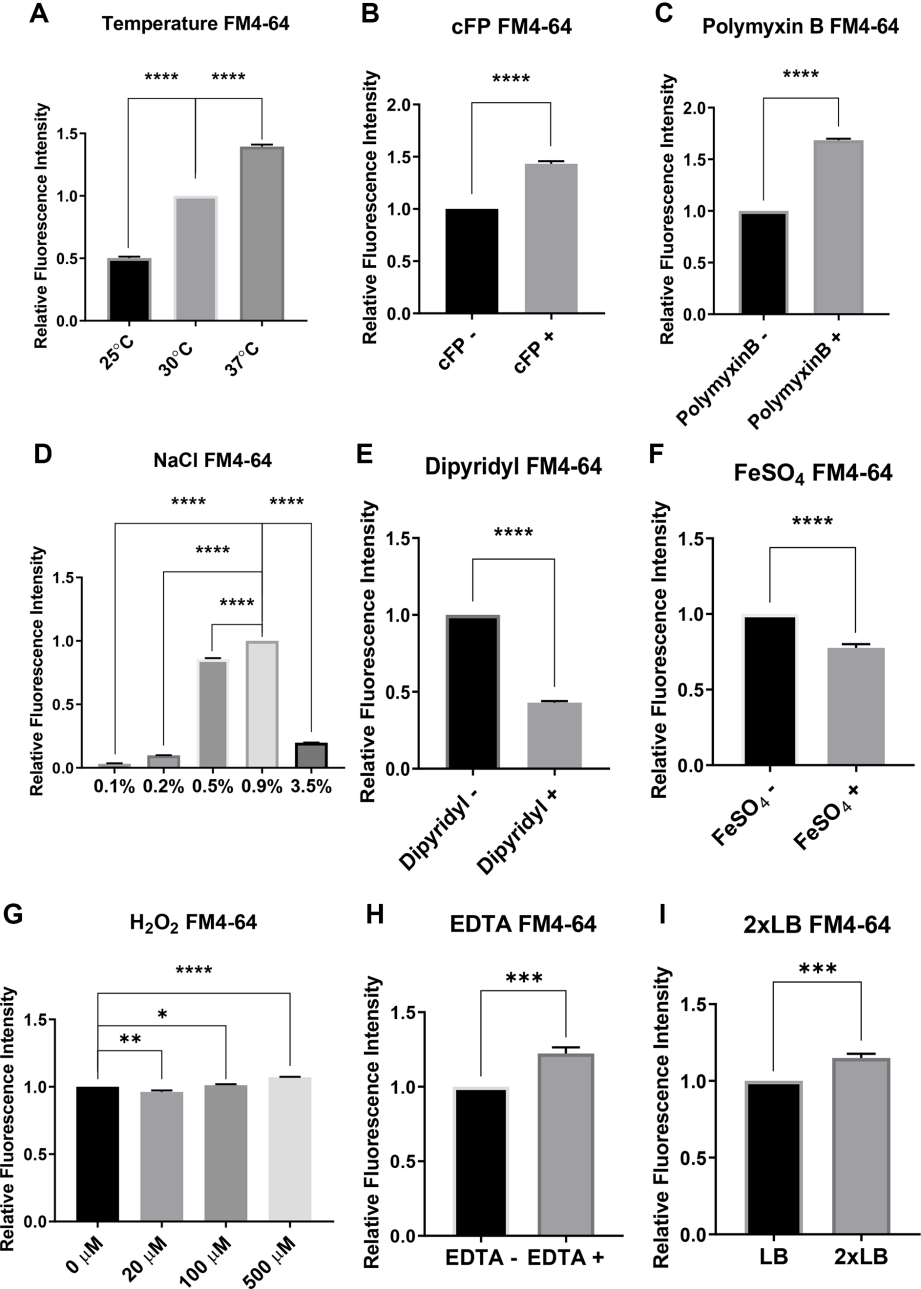
Effects of various physical and chemical factors on the BEV production of *V. vulnificus* as assessed by the FM4-64 dye analysis. (**A**) Effect of three different temperatures. (**B**) Effect of the quorum-sensing signal molecule cFP. (**C**) Effect of the antibacterial agent polymyxin B. (**D**) Effect of various concentrations of NaCl. (**E**) Effect of the iron chelator 2,2'-dipyridyl. (**F**) Effect of FeSO_4_. (**G**) Effect of various concentrations of H_2_O_2_. (**H**) Effect of the divalent ion chelator EDTA. (**I**) Effect of the enriched medium 2 × LB. See the Materials and Methods section for details. The data are average values from three independent experiments, and error bars denote the standard deviations (Student’s *t*-test: *, *p* < 0.0308; **, *p* < 0.0038; ***, *p* < 0.0007; ****, *p* < 0.0001).

Effect of temperature on the BEV generation varies from species to species [23]. In *E. coli*, the amount of vesicles increases when the temperature increases, and in *P. aeruginosa*, the amount of vesicles does not change significantly even when the temperature increases. In some species, the amount of vesicles increases at low temperatures [8, 24, 25]. However, in *V. vulnificus*, it has not yet been examined how the BEV production is affected by temperature. To examine the effect of temperature on the amount of BEV production, *V. vulnificus* was grown at 25°C, 30°C, and 37°C, and supernatants of the cell cultures were separated at 1-h intervals to perform western hybridization using antibody against OmpU to estimate the amount of BEV production. It appears that the amount of BEVs is proportional to the growth of cells, and the effect of temperature on the amount of BEVs was rather due to the effect on growth of cells (Fig. S3). FM4-64 showed that the level of the generation of BEVs was significantly higher at 37°C than at 25°C or 30°C (Fig. 1A).

### b. Quorum-Sensing Signal Molecule cFP

It has been reported that the *Pseudomonas quinolone* quorum-sensing signal molecule produced by *P. aeruginosa* stimulates BEV formation [26]. *V. vulnificus* employs cFP as a quorum-sensing signal molecule, which plays an important roles associated with the pathogenicity [17]. We assessed the effect of cFP on the production of BEV in the cognate cells. *V. vulnificus* cells grown with 5 mM cFP or with DMSO as control at 30°C were collected at 1-h intervals. cFP was treated when the optical density of cells at 600 nm reached to 0.3. cFP slightly retarded the growth cells (Fig. S4), but significantly increased the generation of BEVs (Fig. 1B).

### c. Antimicrobial Agent Polymyxin B

The antimicrobial peptide, polymyxin B, has been known to make pores or modify membrane fluidity by acting on the outer membrane, and BEV in *Escherichia coli* acts as a decoy against polymyxin B, resulting in resistance to the antibiotic [9]. To test the possibility that polymyxin B affects the BEV generation in *V. vulnificus* cells, 20 μg/ml of polymyxin B was treated and the BEV productions at various growth time points were assessed. Polymyxin B retarded the growth of cells (Fig. S5), but significantly increased the generation of BEVs (Fig. 1C).

### d. Sodium Chloride

It has been reported that BEV release is increased under osmotic pressure in *P. putida*, but, in the case of *E. coli*, a mutation in *ompR* regulating osmotic pressure does not significantly affect BEV production [18, 28]. These reports led us to examine the possibility that osmotic pressure may affect the BEV production in *V. vulnificus*. We culture the cells under different concentrations of NaCl, and cell growth and the BEV productions were assessed. Both cell growth and the BEV production was maximal at the physiological NaCl concentration (0.9%), and hypertonic or hypotonic concentrations significantly decrease both cell growth (Fig. S6) and the BEV production (Fig. 1D).

### e. Iron Chelating Agent 2,2'-Dipyridyl

We also compared cells grown in LB and cells grown LB with the iron chelator 2,2'-dipyridyl. The addition of the chelator significantly decreased the cell growth (Fig. S7). The production of BEV was also significantly decreased in the presence of 2,2'-dipyridyl (Fig. 1E).

### f. Iron Ion

Previous study showed that the effect of iron ion on BEV production varies among bacterial species [29]. To examine the effect of iron ion on *V. vulnificus*, cells were grown in LB and LB with additional 25 μM of FeSO_4_, cell growth and BEV production was compared. Addition of FeSO_4_ did not significantly affect both cell growth (Fig. S8) but the BEV production was significantly decreased by the additional FeSO_4_ (Fig. 1F).

### g. Hydrogen Peroxide

Oxidative stress has been reported to affect the OMV production in *Nisseria meningitidis* or *Campylobacter jejuni* [30, 31]. We examined the effect of hydrogen peroxide at various concentrations on the BEV production in *V. vulnificus*. Cells grown in LB were treated with 20 μM, 100 μM, or 500 μM of H_2_O_2_ and DIW as control, and the BEV production was measured. Cell growth was gradually retarded as the concentration of H_2_O_2_ was increased, and, at 500 μM, cell growth was significantly retarded in the experiment conditions (Fig. S9). However, production of BEVs did not exhibit significant differences among these concentrations of H_2_O_2_ in *V. vulnificus*. When considering the relatively values from the FM4-64 assay, it was observed to increase significantly with higher concentration of H_2_O_2_ (Fig. 1G). However, this increase in value appears to be primarily due to a lower number of cells. Therefore, the effect of H_2_O_2_ on the generation of BEVs does not appear to be significant.

### h. EDTA

It has been known that treatment with the chelating agent ethylenediaminetetra-acetic acid (EDTA) can increase the release of BEVs by trapping cations and disrupting membrane stability [32, 33]. To examine if that is also the case in *V. vulnificus*, cells grown with or without 100 μM of EDTA were collected at 1-h interval growth time points, and assessed the level of BEV generation. EDTA slightly retarded the cell growth (Fig. S10). The production of BEVs at early growth stage was not affected by EDTA, but at late growth stage, EDTA significantly enhanced the BEV production. The FM4-64 assay also showed that the EDTA treatment significantly increased the BEV production (Fig. 1H).

### i. High Concentration of Rich Medium

The BEV production has been reported to be affected by the composition of culture media [34, 35]. We compared the BEV productions between cells grown in a regular Luria-Bertani medium (Difco, USA) and cells grown in modified Luria-Bertani broth with strengthened nutrition (2 × LB; tryptone 20 g/l, yeast extract 10 g/l, NaCl 10 g/l). Cells were collected at 1-h intervals and the BEV productions were compared between these two groups. As shown in Fig. S11, cells grow faster in 2 × LB, and the production of BEV was significantly increased in the medium (Fig. 1I).

**Fig. 2:**
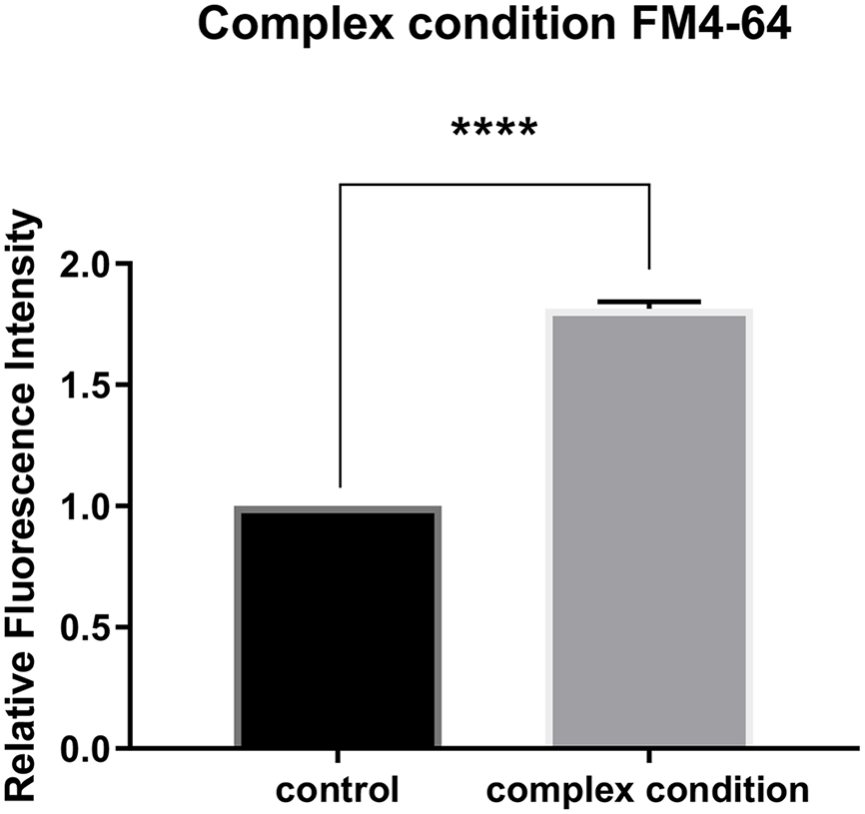
Effects of the optimized conditions on the BEV production of *V. vulnificus* as assessed by the FM4-64 dye analysis. (**A**) Effect on the BEV production of *V. vulnificus* cells cultured at 37°C in the 2 × LB medium in the presence of EDTA. See text for details. The data are average values from three independent experiments, and error bars denote the standard deviations (Student’s *t*-test: ****, *p* < 0.0001).

The conditions which enhance the BEV production are summarized as follows: growth at 37°C, addition of cFP, addition of EDTA, addition of polymyxin B, physiological NaCl concentration (0.9%), and using 2LB as a culture medium. Among these conditions, polymyxin B harms to cells and cFP is not readily affordable in large amount for its expensive commercial price. Therefore, without these two chemicals, we applied remaining optimal conditions for the maximal BEV production to culture cells. At these optimal conditions for the BEV production of *V. vulnificus*, cell growth was not discernably different from that at regular growth condition (grown in LB at 30°C without EDTA) (Fig. S12A). However, under the optimal conditions, we were able to obtain a significantly larger amount of BEVs, as confirmed by both western hybridization (Fig. S12B) and FM4-64 fluorescent dye analysis (Fig. 2). The FM4-64 assay indicated that these conditions resulted in approximately a 70% increase in BEV production compared to the regular condition. Therefore, we employed these combination of conditions for the harvesting BEVs from *V. vulnificus* for the subsequent experiments.

### DLS Analysis of BEVs Obtained under the Optimal Conditions

**Fig. 3:**
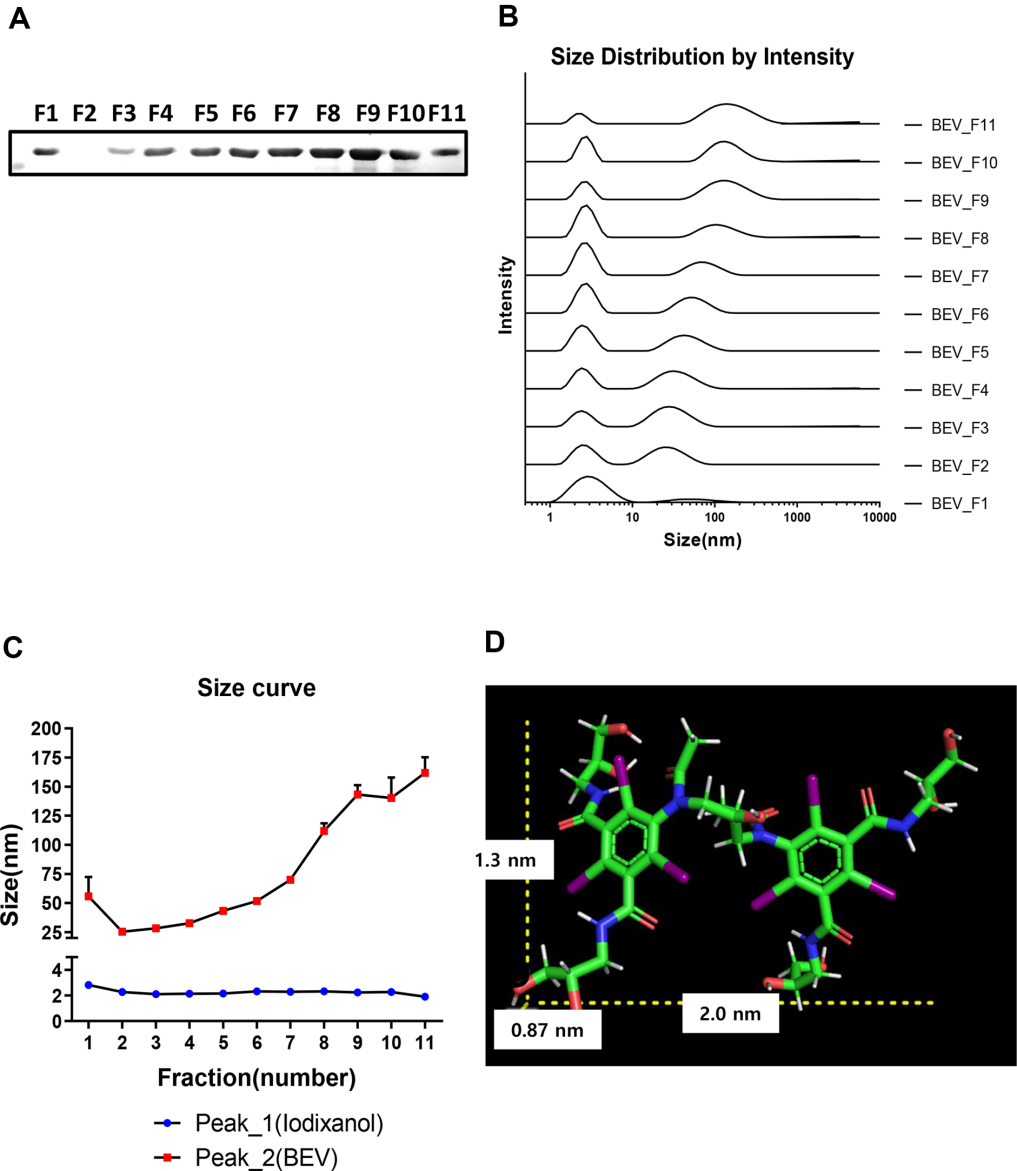
DLS measurement of BEVs separated by density gradient ultra-centrifugation. (**A**) Western hybridization analysis of BEV in each fraction after density gradient ultracentrifuge. These fractions were designated as F1 through F11 from the bottom of the tube to the top. (**B**) DLS analysis of the fractions after density gradient ultracentrifuge. Each curve was intensity-weighted distribution. (**C**) Average sizes of BEVs in fractions. (**D**) Computer program-generated image of the structure of iodixanol using the Pymol program. It is estimated that the size of the molecule is approximately 0.87 × 1.3 × 2.0 nm.

To obtain large amount of BEVs from *V. vulnificus* MO6/24-O, cells were grown in the optimal conditions described above for 8 h, and BEVs were purified by the density gradient ultracentrifugation as described in the Materials and Methods section. Total 11 fractions with the 1 ml volume each were obtained after density gradient ultracentrifugation. The first fraction obtained from the bottom of the tube was named F1, and the last layer obtained at the top as F11. The amount of BEVs in these fractions were quantitatively estimated by western hybridization using antibodies against OmpU (Fig. 3A). A band in F1 may be precipitates of debris of BEVs. Bands were detected from the F3. The dynamic light scattering (DLS) analysis showed that there appear two peaks in all fractions. Intensity-weighted size distribution of each fraction is shown in Fig. 3B. Each fraction has two peaks, and the first peak between 1 and 10 nm represents iodixanol. As the average size of each fraction is considered, the size tends to be smaller in the lower layer with higher density and increases in size as the density decreases. The diameter of BEVs appears to be a minimum value of 25.44 nm (F3) and a maximum value of 161.7 nm (F11)(Fig. 3C). The size of iodixanol estimated by Pymol is 2.0 nm × 1.3 nm × 0.87 nm (Fig. 3D).

### NGS Analysis of sRNAs in Cell and in BEVs

We conducted an analysis of small RNAs (sRNAs) present both in *V. vulnificus* cells and in their BEVs. To achieve this, sRNAs were meticulously isolated from cells and BEVs, followed by the preparation of cDNA libraries, employing the methods outlined in the Materials and Methods section.

**Sequencing and initial data assessment:** TruSeq (Illumina) was performed, employing paired-end reads with a read length of 101 base pairs (bp). The total read bases amounted to 1,797,077,749 for the cell extract and 1,811,121,597 for BEVs. In terms of the total number of reads, the cell extract and BEVs yielded 17,792,849 and BEV 17,931,897, respectively.

**Trimming process and quality control:** The smRNA-Seq Kit Manual guidelines were followed for the trimming process, utilizing Cutadapt [36]. Reads, retained if they were a minimum of 10 nucleotides (nt) in size, and the adapter sequence was removed, which was defined by initial three nucleotides of the reads and a 5'-AAAAAAAAAA-3' adapter sequence. Any reads containing 'N' (ambiguous read) bases were omitted. Additional criteria of a minimum overlap and quality cutoff of 10 were imposed. In the cellular extract, out of the initial read count, 17,392,611 reads (97.8%) contained adapters, while 507,291 reads (2.9%) were deemed too short, and 1,462 reads (<0.0%) possessed excessive ‘N’ bases. Consequently, 17,284,096 (97.1%) reads passed the filters resulting in a total of 693,373,933 bp (38.6%). In the BEVs, out of the initial count, 16,805,457 reads (93.7%) passed through the filters, 1,909,342 reads (10.6%) were classified as too short, and 7,535 reads (<0.0%) contained excess of ‘N’. Therefore, 16,015,020 reads (89.3%) passed the filter process, amounting to a total 555,284,653 bp (30.7%).

**Fig. 4:**
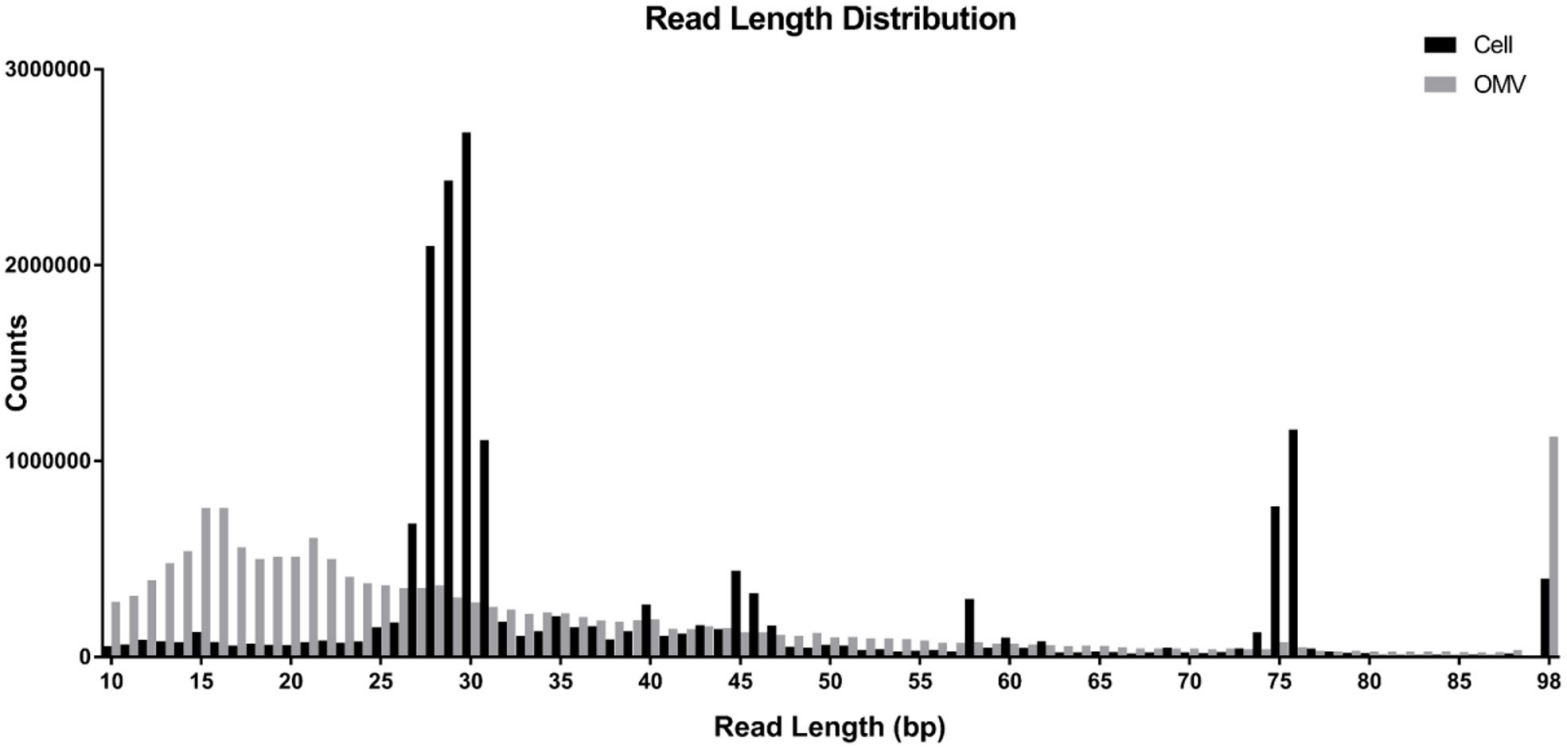
Nucleotide length distribution from cells and BEVs. Nucleotide length distribution of reads after trimming. X-axis starts from 10 bp to 98 bp. The black columns represent counts from cells, and the gray columns represent counts from BEVs.

**Data analysis:** A post-trimming length distribution analysis of reads revealed that the predominant sRNA sizes in BEVs range from 27 to 30 nucleotides. Conversely, sRNA sizes within cells were more broadly distributed, indicating that sRNA encapsulated in BEVs are notably shorter than those within the cell (Fig. 4). Genome mapping was utilizing the NCBI Assembly ASM18658v1 with Bowtie2 [37]. Out of the 17,284,096 reads within the cell, 16,632,806 (96.2%) were aligned to the genomic library, meanwhile only 6,089,479 (38.0%) reads from BEV were aligned, highlighting discrepancy in the alignment success rate between the cell extracts and BEVs.

### Comparative Analysis of Expression of sRNA with Known Functions in Cells and BEVs.

In earlier research, functional sRNAs have been identified in various *Vibrio* species including *V. cholerae*, *V. fischeri*, *V. parahaemolyticus*, and *V. alginolyticus* [38-51]. The sequences of homologs for these sRNAs were predicted through NCBI Blast search [52] to map the start and end sequences on the genome of *V. vulnificus*, and the number of reads mapped to the corresponding positions are shown in Table 1.

We conducted a comparison of the relative ratio of read numbers of each of these sRNA between cell and BEVs. Under the assumption that sRNA packaging into BEVs would directly correspond to the abundance of these sRNAs in cells, one would expect consistent ratios for each sRNA across both environments. However, our findings, as presented in Table 1, indicated significant variations in these ratios based on the sRNA in question. This suggests that BEVs might preferentially package certain sRNAs.

In an attempt to uncover any sequences indicative of BEVs among the known sRNAs, we compared the sequences of sRNA homologs that were predominantly found in BEVs. Despite out rigorous analysis, we were unable to discern any sequences characteristic to sRNAs which are dominantly present in BEVs (data not shown).

### A search for Common sRNA Sequences in BEV

In our pursuit to identify any prevalent nucleotide sequences among the sRNAs in BEVs, we ranked the BEV-derived reads based on their count from highest to lowest and closely analyzed the nucleotide sequences on the top 30 entries. The multi-alignments were performed using Clustal Omega revealed occasional short sequences such as 5'-GCTGGCTCCG-3' in a subset of sRNAs (Fig. S13). However, these sequences were limited to only a few sRNA fragments. Additionally, upon further analysis, these sequences appear to be segments of mRNA from coding genes. Consequently, our search did not uncover any significantly prevalent nucleotide patterns or sequences that could be characterized as common among the BEV sRNAs.

## Discussion

Various biological roles of BEVs have been emerged by recent progress in the related studies. Especially the role as a tool for a delivery system of macromolecules is attractive for genetic and bioengineering fields [53]. We assumed that BEVs can be utilized for the lateral transfer of sRNAs to other cells including bacterial and eukaryotic cells, which can be utilized to manipulate physiology of recipient cells by modulating the expression of certain functions. This study aims at the acquisition of basic information on optimal conditions for the production of BEVs in the pathogen, and that on natures of sRNAs packaged in them as well.

Not much information is available for the production of BEVs in the human pathogen *V. vulnificus*. We tested chemical and physical conditions which have been reported to affect the production of BEVs in other bacterial species. Among examined conditions, we observed that high temperature (37°C), addition of EDTA, cFP, and polymyxin B, and culture in 2 × LB turn out to result in significantly high amount of BEVs. The addition of iron did not significantly affect the production of BEVs. However, addition of the iron chelator 2,2'-dipyridyl diminished the production. These results suggested that the minimal amount or iron present in rich medium is enough for the BEV production, and additional amount does not result in improved result. However, depletion of iron is adverse for the BEV production. Among these conditions, cFP and polymyxin B are excluded for the further studies. Polymyxin B may harm to the growth of cells. When we consider the utilization of BEVs as a tool for the lateral transfer of molecules, this compound may give a detrimental effect on physiology of donor cells. Even though cFP has a positive effect on the BEV production, the compound is required for a large amount for the mass-production of BEVs such that it is economically disadvantageous due to its high cost. Apparently, combinations of conditions we set up results in significantly higher amount of the BEV production, no matter what the underlying mechanisms would be. Using the conditions, we performed the subsequence studies.

The sizes of BEVs we isolated range between approximately 25 nm and 160 nm in diameter. Previous report showed that OMVs isolated from the wild type V. vulnficus CMCP6 strain is approximately 45 nm in size [54]. This suggests that the procedure we employed may result in the isolation of various forms of BEVs.

We characterized sRNA molecules packed in BEVs and compared them with bacterial cellular sRNAs. We assumed that there exist a certain features specific to sRNAs in BEVs comparing to those in cells. The sizes of sRNAs in BEVs are significantly shorter than those in cells. We do not understand a mechanism behind this phenomenon. The BEVs we purified may be a mixture of at least three different types generally found in gram-negative bacteria as mentioned in the Introduction section. Depending on the types, it is expected to have different mechanisms of sRNA packaging. For instance, in OMVs, sRNA should be passed across the inner membrane and captured in the periplasmic space before the generation of OMVs. At the moment the mechanisms of transport of sRNAs across the inner membrane remains to be studied, but it is likely that smaller molecules can more readily pass across the inner membrane. Meanwhile, EBEVs and OIMVs, sRNA molecules may be captured by chances after cells are ruptured. It is possible that smaller in the size sRNAs would be easier to be captured by just simple diffusion. Another possibility is, even if less likely, a biased procedure for preparation of RNA molecules from BEVs. We followed a standard protocol suggested by manufacture to prepare RNA from BEV. For some unknown reasons, smaller RNA molecules may be prepared preferentially. Precise mechanism waits for more detailed studies. We also tried to find specific nucleotide sequences found in sRNAs in BEVs. If there exist such sequences, it may be feasible to design sRNA molecules which can be more easily packed in BEVs. However, our results showed that there is no such sequence motif clearly found in sRNA candidate molecules in BEVs. When we compared the ratio of biologically studied sRNAs between cell and BEVs, the ratios of them varies to a large extent. Furthermore, sRNAs preferentially more packaged (or less packaged) do not have a clear tendency in their length or nucleotide sequences. These may suggest that the package of sRNAs into BEVs is not a random process but there may exist an unknown mechanism. For instance, certain machinery may be involved in the process.

It is possible that, in nature, pathogens may disseminate BEVs harboring various functions to neighboring cells with the same species in community. Furthermore, BEVs may also be fused to microorganisms of different species to deliver certain functions to affect microbiota. The BEVs may also be fused to host cells to deliver functions, and, thereby, affect their biology. Researches on BEV are at a beginning stage, and, especially for *V. vulnificus*, virtually nothing has been reported on BEVs. This study will be served to provide a basic information on BEV of the human pathogen *V. vulnificus*.

## Supplemental Materials

Supplementary data for this paper are available on-line only at http://jmb.or.kr.



## Figures and Tables

**Table 1:** Expected start and end sequences of sRNA homologs on the genome of *V. vulnificus* and their average read depth.

sRNA homolog	Related species*	NCBI Accession No.	Average read (OMV)	Average read (Cell)	Ratio (OMV/Cell)
MicV	*V. cholerae*	NZ_CP047295	53	37791	0.00140
VqmR	*V. cholerae*	NZ_CP047296	59	38224	0.00154
Qrr4	*V. vulnificus*	NC_014966	18	1815	0.00997
Qrr1	*V. vulnificus*	NC_014965	2	200	0.01000
Qrr3	*V. vulnificus*	NC_014966	63	5368	0.01174
VadR	*V. cholerae*	NZ_CP047296	241	11967	0.02014
GcvB	*V. alginolyticus*	NZ_CP016224	108	4164	0.02594
MicX	*V. cholerae*	NC_002506	32	755	0.04238
Qrr5	*V. vulnificus*	NC_014966	16	376	0.04255
VrrA	*V. cholerae*	NZ_CP024867	34	619	0.05493
TfoR	*V. cholerae*	NZ_AP018677	66	952	0.06933
MtlS	*V. cholerae*	NC_002506	178	2457	0.07245
CsrC	*V. cholerae*	NZ_CP047295	170	2137	0.07955
Qrr2	*V. vulnificus*	NC_014966	16	148	0.10811
CsrD	*V. cholerae*	NZ_CP047295	45	410	0.10976
CsrB	*V. cholerae*	NZ_CP047295	125	1119	0.11171
FarS	*V. cholerae*	NZ_CP047295	40	269	0.14870
SsrS	*V. fischeri*	NC_006840	774	2553	0.30317
RnpB	*V. fischeri*	NC_006840	1184	2013	0.58818
SsrA	*V. fischeri*	NC_006840	8106	8268	0.98041
Spot-42	*V. parahaemolyticus*	NC_004603	501	116	4.31897
PsrN	*V. fischeri*	CP000021.2	609	26	23.4231
RyhB	*V. fischeri*	CP000020.2	306	6	51.0000

*Certain sRNA sequences in the *V. vulnificus* genome have not been assigned the accession numbers. For these sequences, we have instead cited the accession numbers of homologous counterparts of related species.
