# Supplementary material for: Optimizing Conditions for the Production of Bacterial Extracellular Vesicles of Vibrio vulnificus and Analysis of the Inner Small RNA Compositions
Source: J Microbiol Biotechnol. 2023 Dec 5;34(1):29–38. doi: 10.4014/jmb.2310.10002 (PMC10840491; doi:10.4014/jmb.2310.10002)
Supplement: Supplementary file 1 [file jmb-34-1-29-supple.pdf]

J. Microbiol. Biotechnol.
https://doi.org/10.4014/jmb.2310.10002

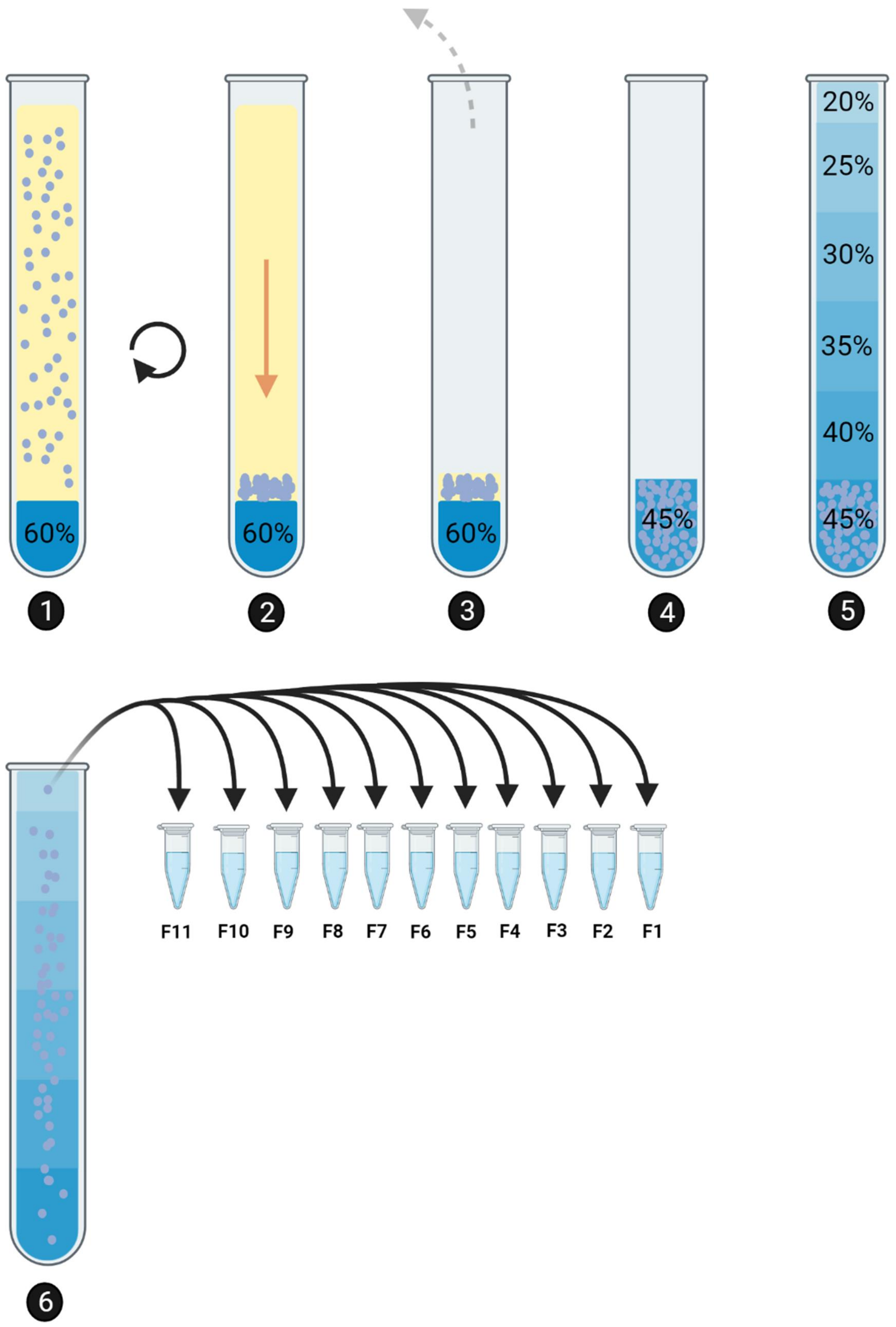

Supplementary Fig. S1. Workflow of density gradient ultracentrifugation for the isolation of BEVs.

Step 1: A preparation containing 60% iodixanol is layered beneath the supernatant, with the dots representing the BEVs. Step 2: After ultracentrifugation, BEVs accumulate in a defined layer between the iodixanol and the supernatant layers. Step 3: After the first ultracentrifugation, most of the supernatant is discarded, leaving only a portion that contains the BEVs. Step 4: The remaining supernatant is then mixed with 60% iodixanol in a 1:3 ration, resulting in a 45% solution. Step 5, This step involves creating a density gradient by layering solutions of different concentrations. Finally, in step 6, ultracentrifugation is performed again. After this step, each fraction is collected for further analysis. The 'Materials and Methods' section provides detailed descriptions of the procedure.

```python
import os
import shutil
import pandas as pd
from Bio import SeqIO

def get_gene_info(gb_file):
    gene_info = []
    for record in SeqIO.parse(gb_file, "genbank"):
        for feature in record.features:
            if feature.type in ["CDS", "tRNA", "ncRNA", "rRNA","tmRNA"]:
                start = feature.location.start.position
                end = feature.location.end.position
                strand = feature.location.strand
                locus_tag = feature.qualifiers.get("locus_tag", ["N/A"])[0]
                gene = feature.qualifiers.get("gene", ["N/A"])[0]
                product = feature.qualifiers.get("product", ["N/A"])[0]
                gene_info.append((start, end, strand, locus_tag, gene, product))
    return gene_info

def main():
    input_file = "Output/index.txt"
    locations = get_input(input_file)
    gene_info_c1 = get_gene_info("*.gb")
    gene_info_c2 = get_gene_info("*.gb")
    output_file = "Output/output.txt"
    with open(output_file, "w") as f:
        f.write("Name\tLocation\tRead_count_1\tRead_count_2\tGroup_col_num\tLocus_tag\tGene\tProduct\n")
        with open(input_file, "r") as input_f:
            for line in input_f:
                name, location, read_count_1, read_count_2,group_col_num = line.strip().split("\t")
                location = int(location)
                f.write(f"{name}\t{location}\t{read_count_1}\t{read_count_2}\t{group_col_num}\t")
                gene_found = False
                if name=="NC_014965.1":
                    gene_info=gene_info_c1
                else:
                    gene_info=gene_info_c2
                for info in gene_info:
                    start, end, strand, locus_tag, gene, product = info
                    if start <= location <= end:
                        f.write(f"{locus_tag}\t{gene}\t{product}\n")
                        gene_found = True
                        break
                if not gene_found:
                    f.write("N/A\tN/A\tN/A\n")
```

Supplementary Fig. S2. Source code of the Python program.

Source code of the Python program to get position with read counts above a certain value and gene information. As an input file, it has a '.tabular' file obtained through samtools-depth and a sequence file corresponding to chromosome 1 and 2. As an output file, sequence groups having read counts above a certain value, indexes with the first start information of these groups, and related genes are included. See the details in the 'Materials and Methods' section.

```python
def pre_slice():
    df = pd.read_table('*.tabular', header=None)
    filtered_df = df[(df[3] >= 5000)]
    # filtered_df = df[(df[2] >= 5000)]
    start_index = 0
    group_col_num=0
    prev_value = filtered_df.iloc[0, 1]
    for i in range(len(filtered_df)-1):
        before_current_value = filtered_df.iloc[i, 1]
        current_value = filtered_df.iloc[i+1, 1]
        group_col_num=group_col_num+1
        if abs(before_current_value - current_value) >= 10:
            group_df = filtered_df.iloc[start_index:i+1]
            chr_num = filtered_df.iloc[start_index, 0]
            filename = f'group_{chr_num}_{prev_value}_{before_current_value}.txt'
            group_df.to_csv(f'Output/{filename}', sep='\t', index=False, header=False)
            with open("Output/index.txt", 'a') as f:
                f.write(f'{chr_num}\t{prev_value}\t{group_df.iloc[0, 2]}\t{group_df.iloc[0, 3]}\t{group_col_num}\n')
            start_index = i+1
            group_col_num=0
            prev_value = current_value
    group_df = filtered_df.iloc[start_index:]
    filename = f'group_{chr_num}_{prev_value}_{group_df.iloc[-1, 1]}.txt'
    group_df.to_csv(f'Output/{filename}', sep='\t', index=False, header=False)
    with open("Output/index.txt", 'a') as f:
        f.write(f'{chr_num}\t{prev_value}\t{group_df.iloc[0, 2]}\t{group_df.iloc[0, 3]}\t{group_col_num}\n')

def get_input(input_file):
    with open(input_file, "r") as f:
        lines = f.readlines()
        locations = [int(line.split()[1]) for line in lines]
    return locations

if __name__ == "__main__":
    pre_slice()
    main()
```

Supplementary Fig. S2. Source code of Python program. (Continued)

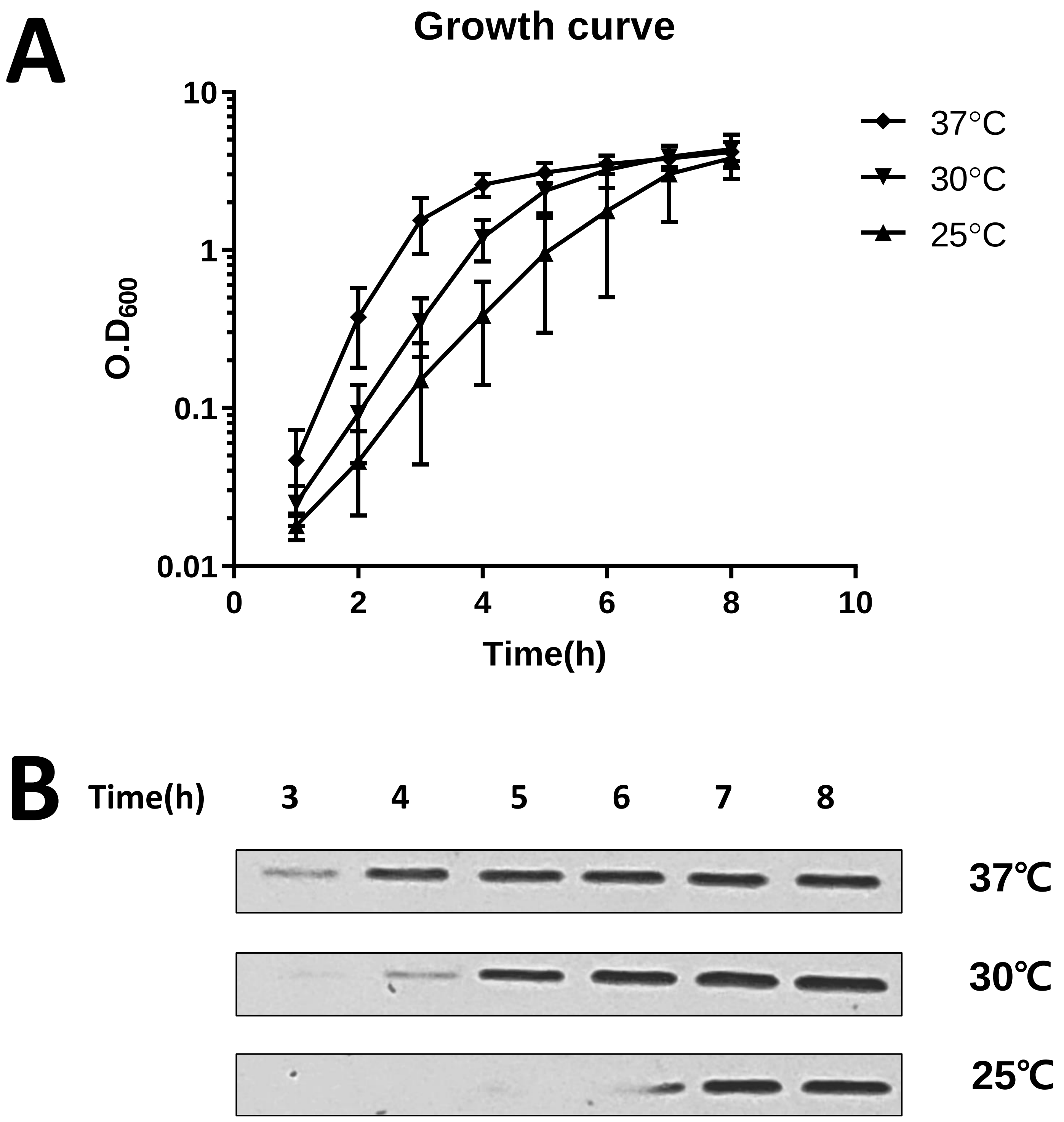

Supplementary Figure S3. Effects of temperature on the growth and BEV production of *V. vulnificus*.

(A) Effect on the growth of cells. The data are average values from three independent experiments, and error bars denote the standard deviations. **(B)** Western hybridization using antibody against OmpU.

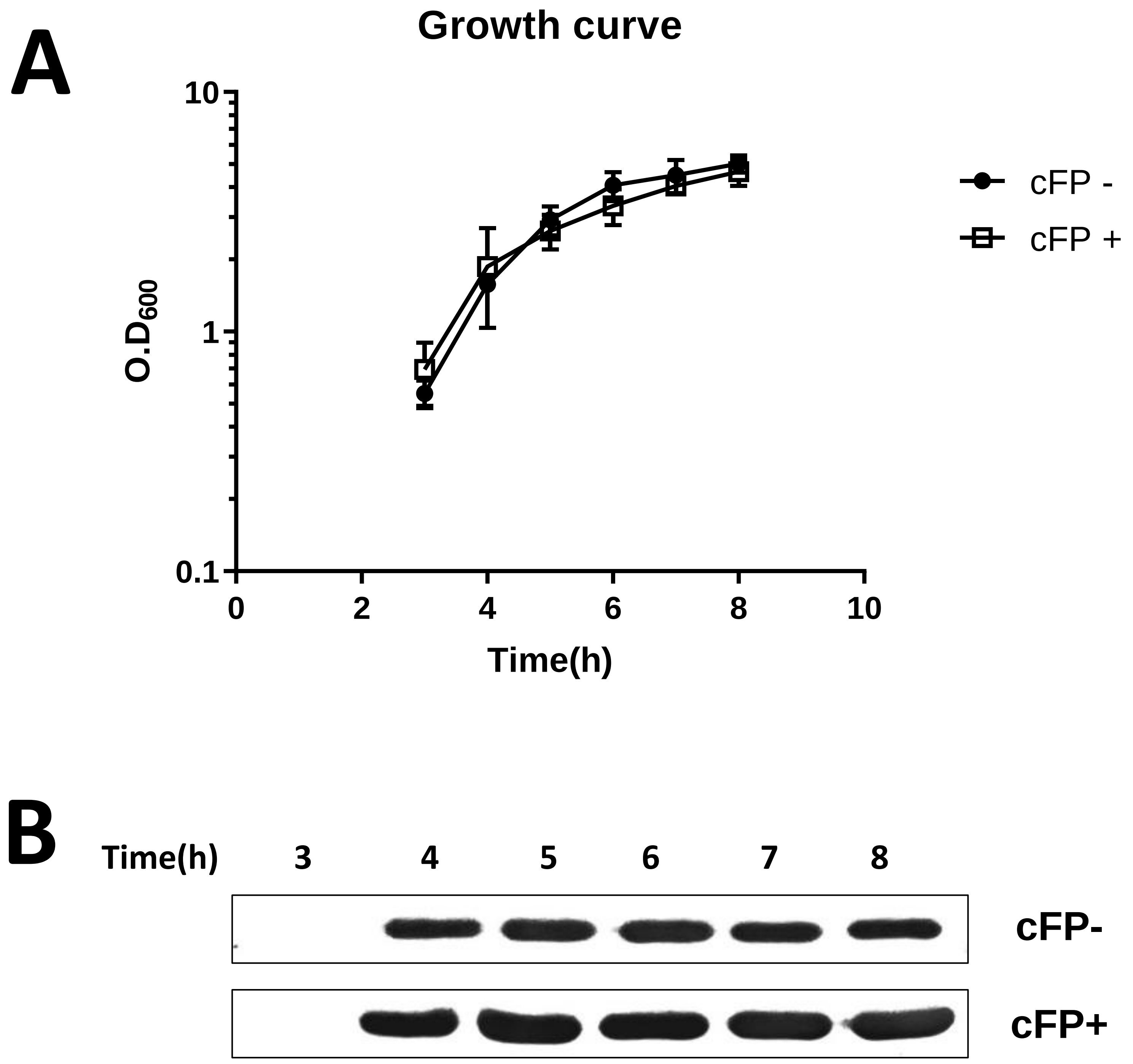

Supplementary Figure S4. Effects of cFP on the growth and BEV production of *V. vulnificus*.

(A) Effect on the growth of cells. The data are average values from three independent experiments, and error bars denote the standard deviations. (B) Western hybridization using antibody against OmpU.

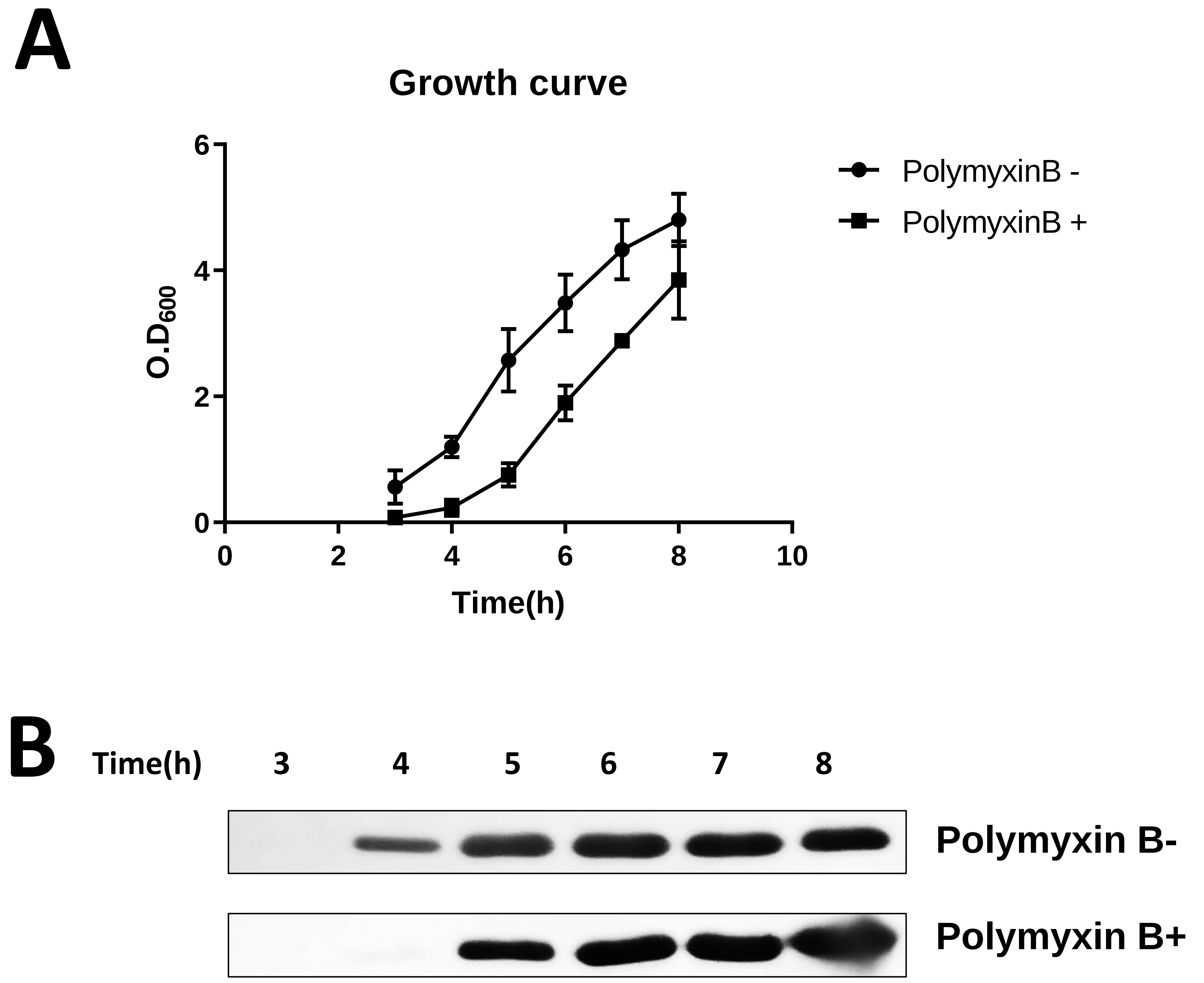

Supplementary Fig. S5. Effects of polynyxin B on the growth and BEV production of *V. vulnificus*.

(A) Effect on the growth of cells. The data are average values from three independent experiments, and error bars denote the standard deviations. **(B)** Western hybridization using antibody against OmpU.

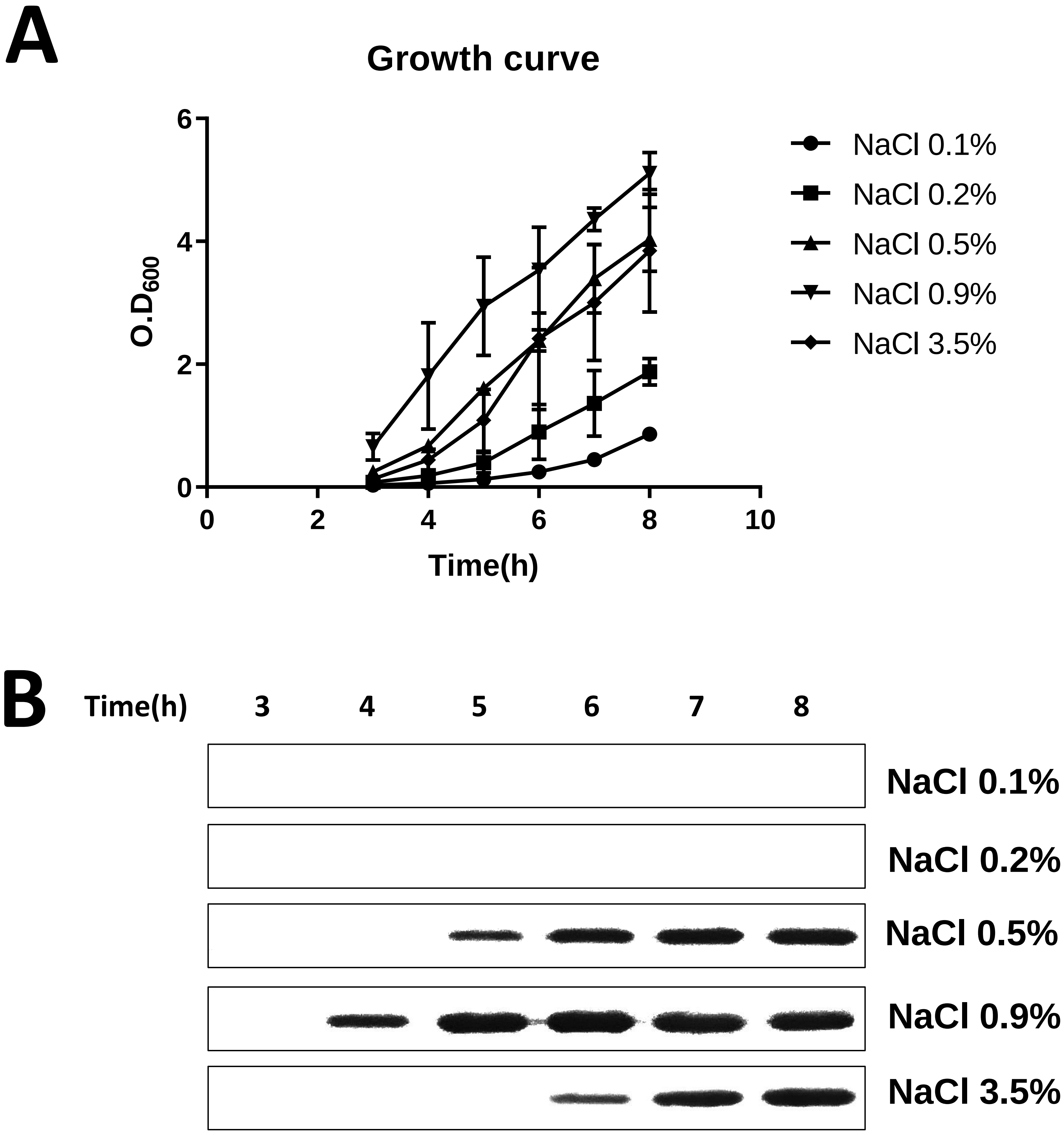

Supplementary Fig. S6. Effects of NaCl on the growth and BEV production of *V. vulnificus*.

(A) Effect on the growth of cells. The data are average values from three independent experiments, and error bars denote the standard deviations. **(B)** Western hybridization using antibody against OmpU.

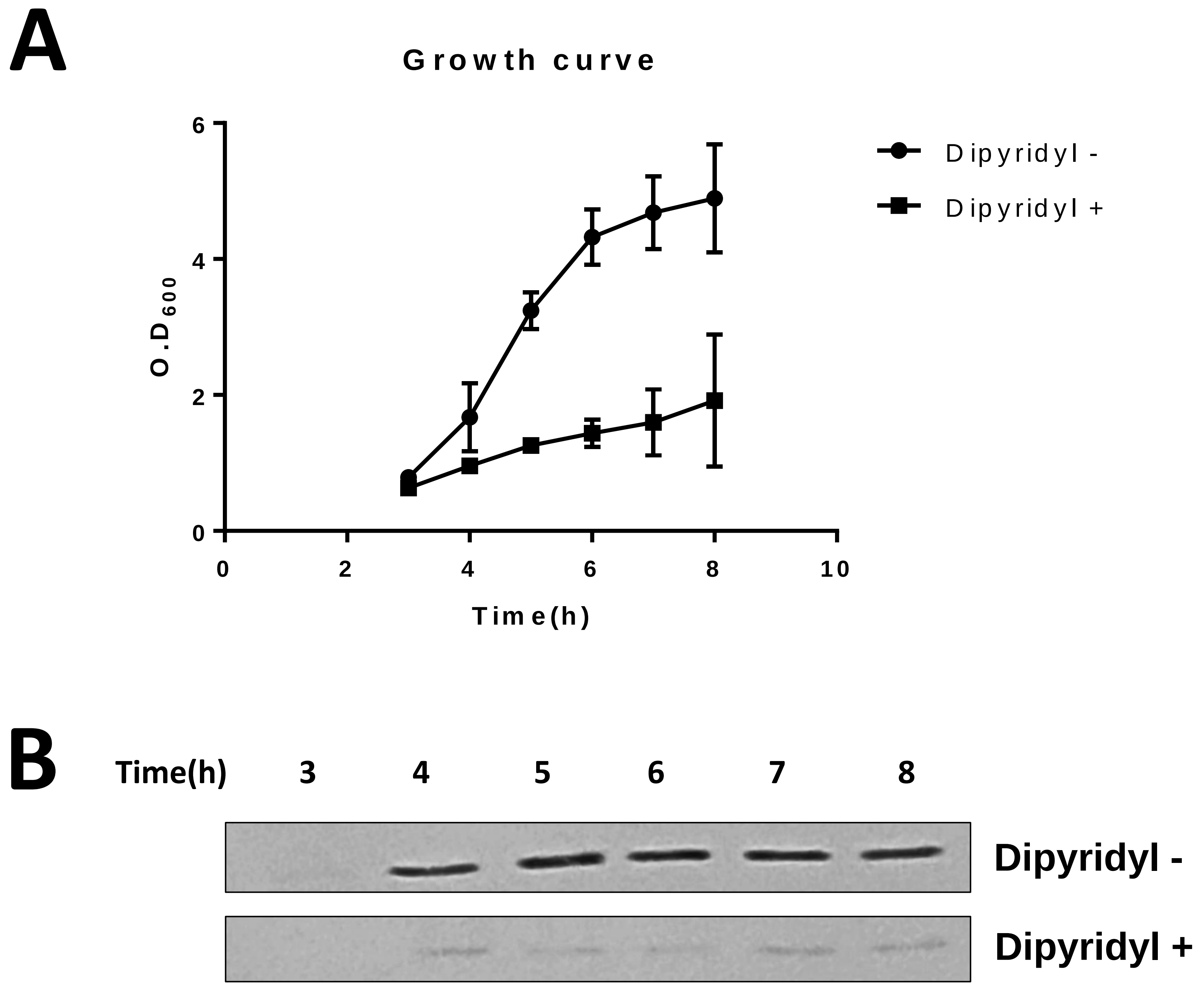

Supplementary Fig. S7. Effects of the iron chelator 2,2′-dipyridyl on the growth and BEV production of *V. vulnificus*.

(A) Effect on the growth of cells. The data are average values from three independent experiments, and error bars denote the standard deviations. **(B)** Western hybridization using antibody against OmpU.

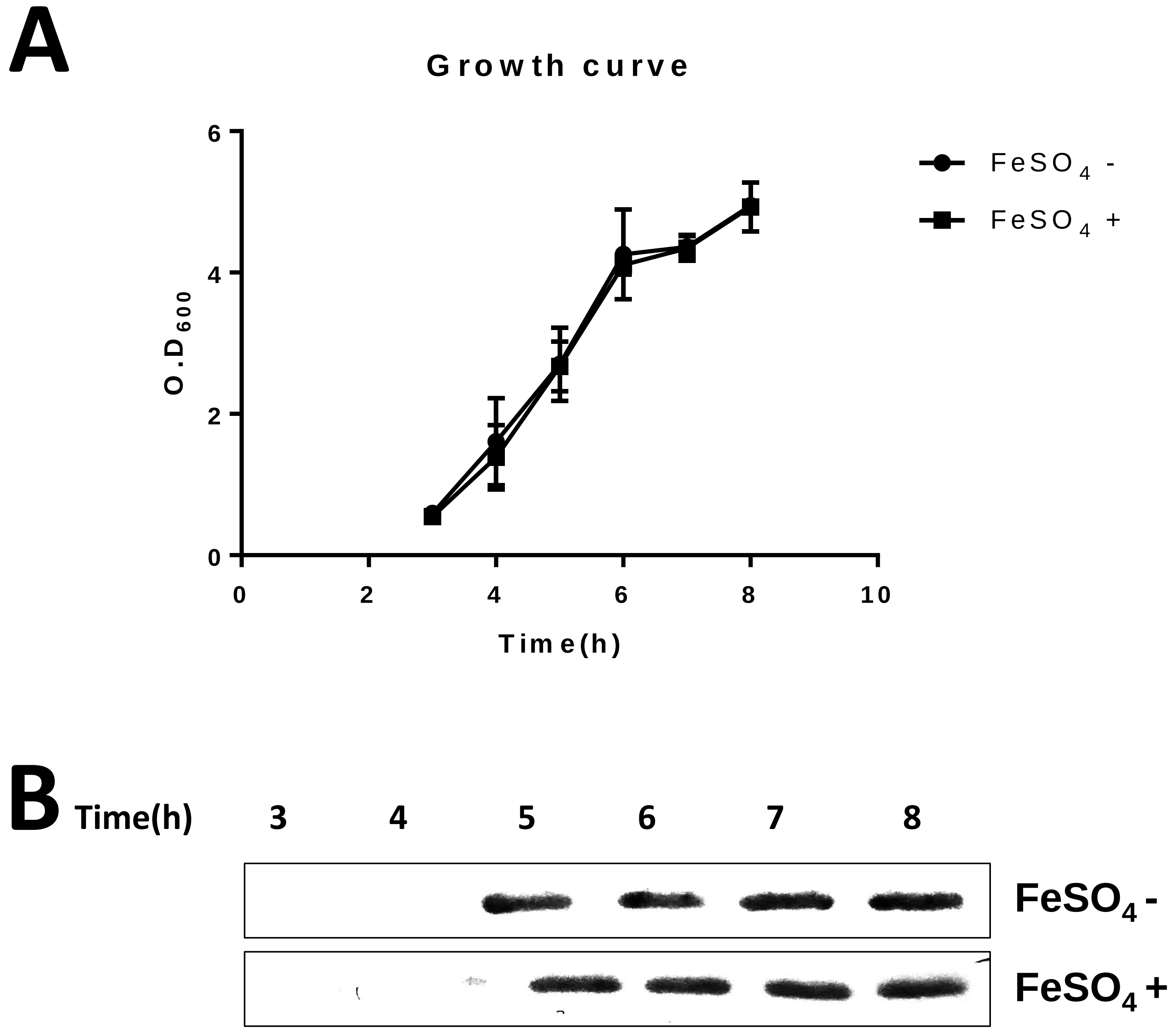

Supplementary Fig. S8. Effects of $FeSO_4$ on the growth and BEV production of *V. vulnificus*.

(A) Effect on the growth of cells. The data are average values from three independent experiments, and error bars denote the standard deviations. **(B)** Western hybridization using antibody against OmpU.

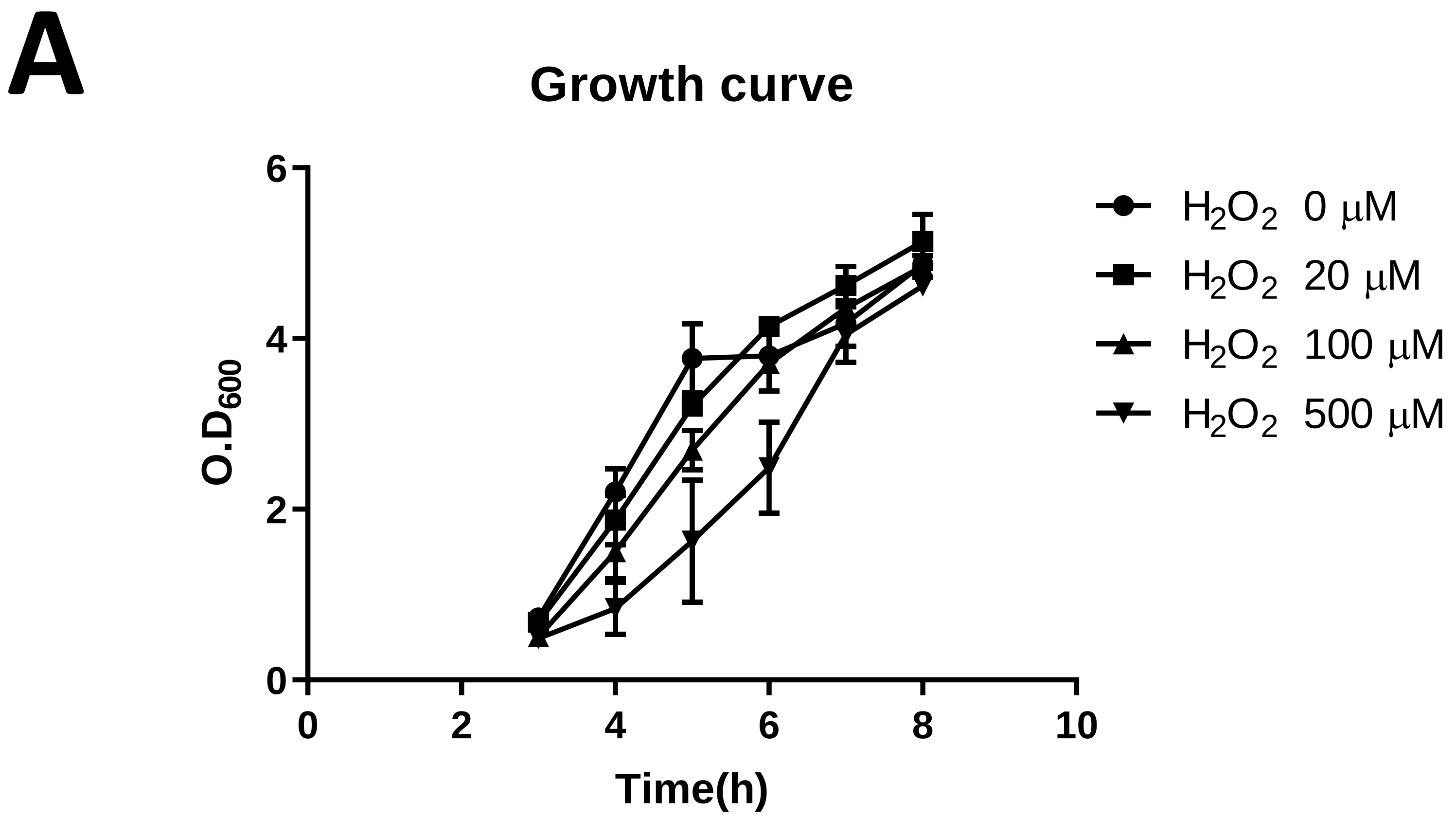

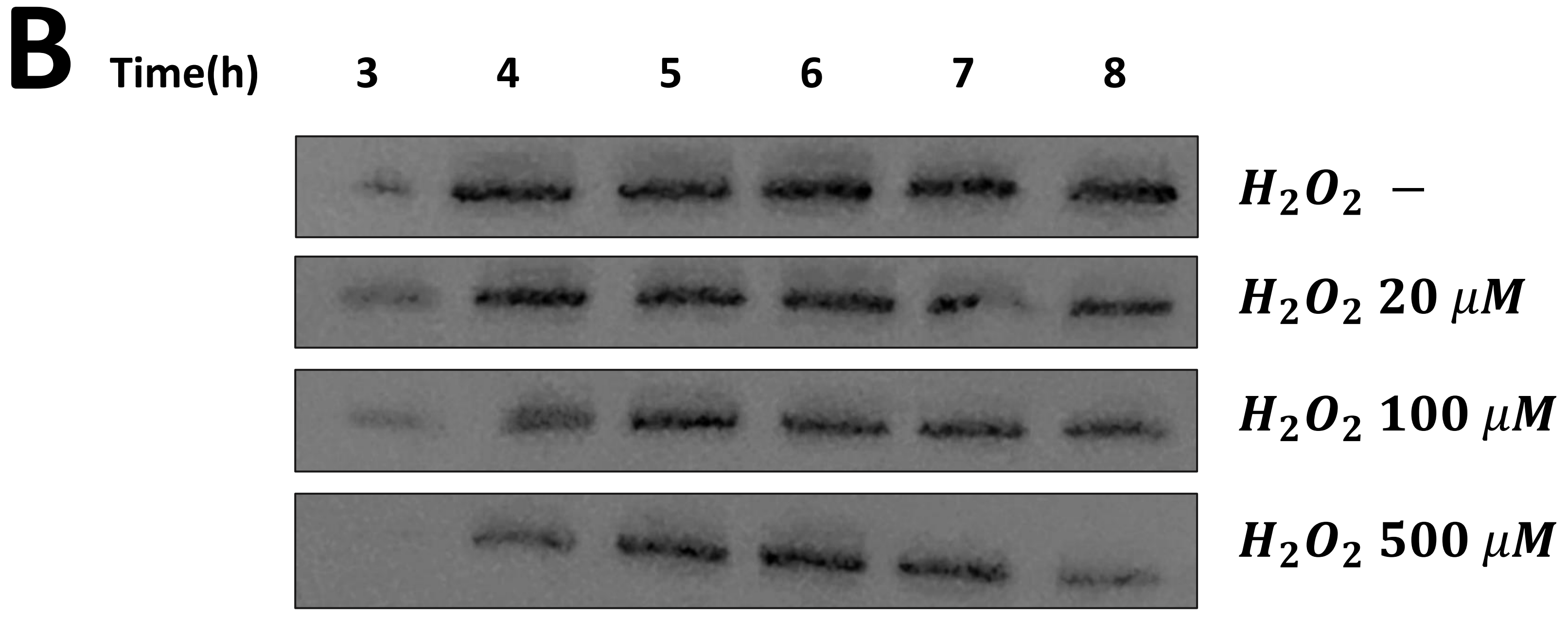

Supplementary Fig. S9. Effects of H_2O_2 on the growth and BEV production of *V. vulnificus*.

(A) Effect on the growth of cells. The data are average values from three independent experiments, and error bars denote the standard deviations. **(B)** Western hybridization using antibody against OmpU.

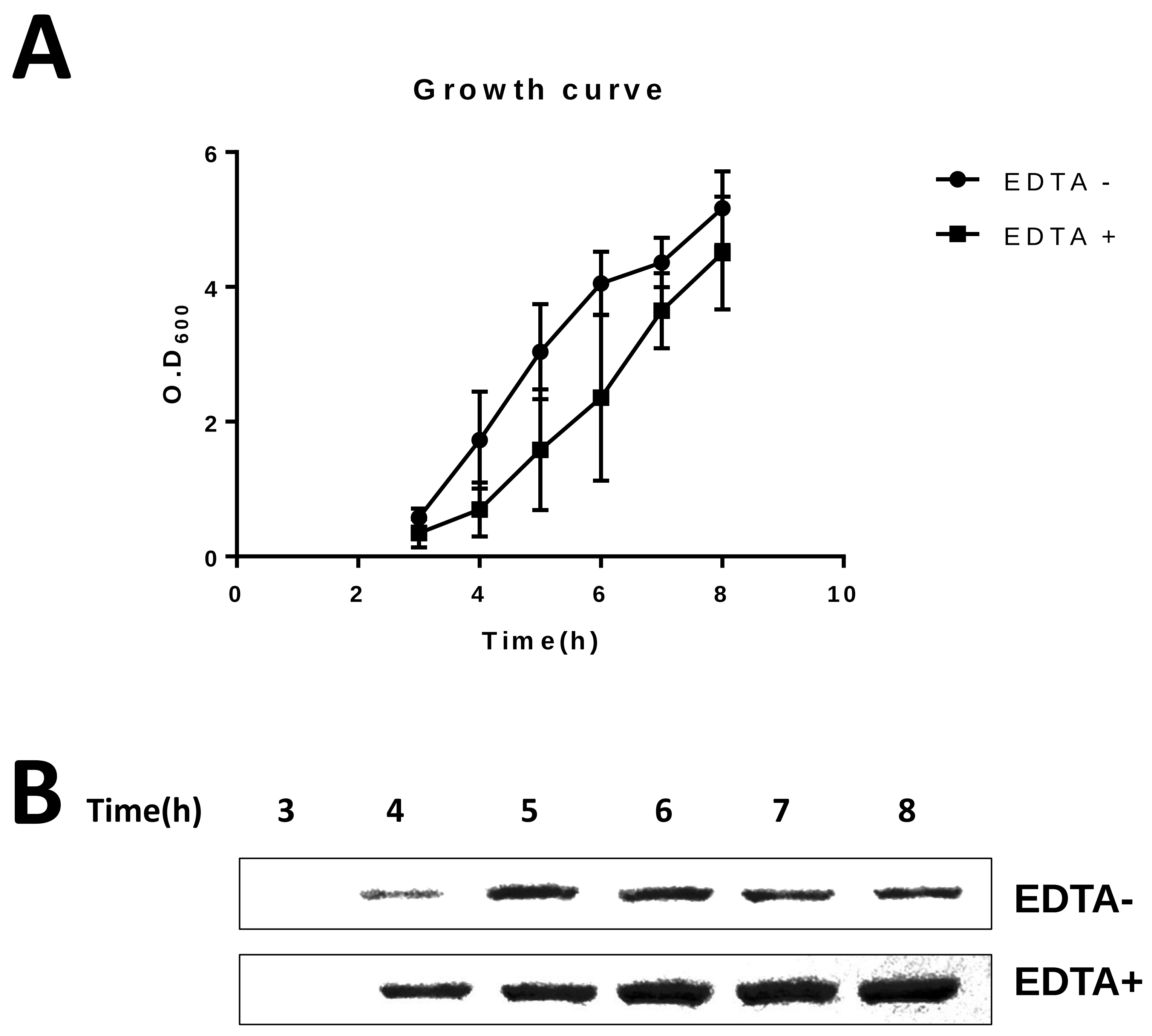

Supplementary Fig. S10. Effects of EDTA on the growth and BEV production of *V. vulnificus*.

(**A)** Effect on the growth of cells. The data are average values from three independent experiments, and error bars denote the standard deviations. **(B)** Western hybridization using antibody against OmpU.

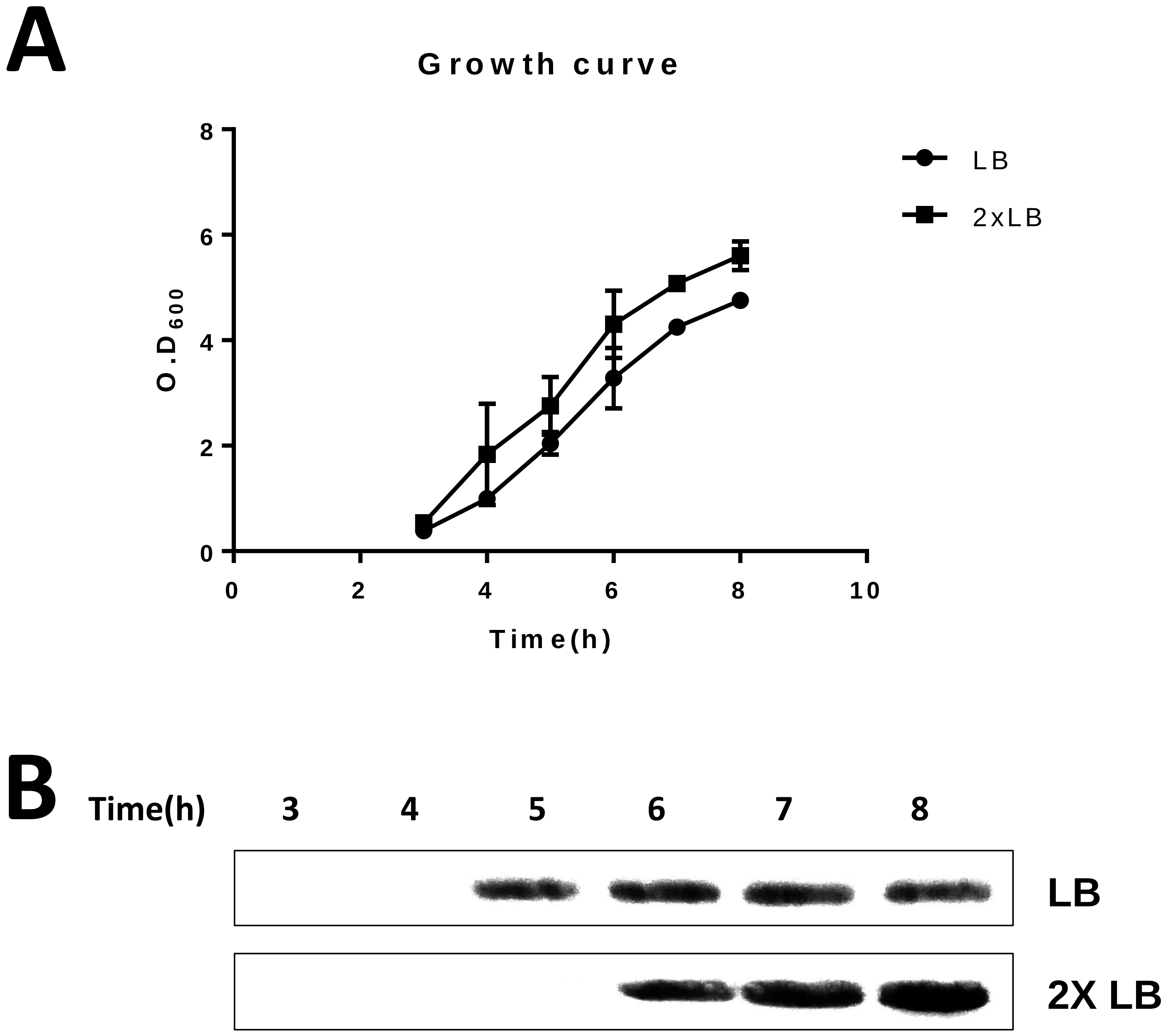

Supplementary Fig. S11. Effects of 2×LB on the growth and BEV production of *V. vulnificus*.

(A) Effect on the growth of cells. The data are average values from three independent experiments, and error bars denote the standard deviations. **(B)** Western hybridization using antibody against OmpU.

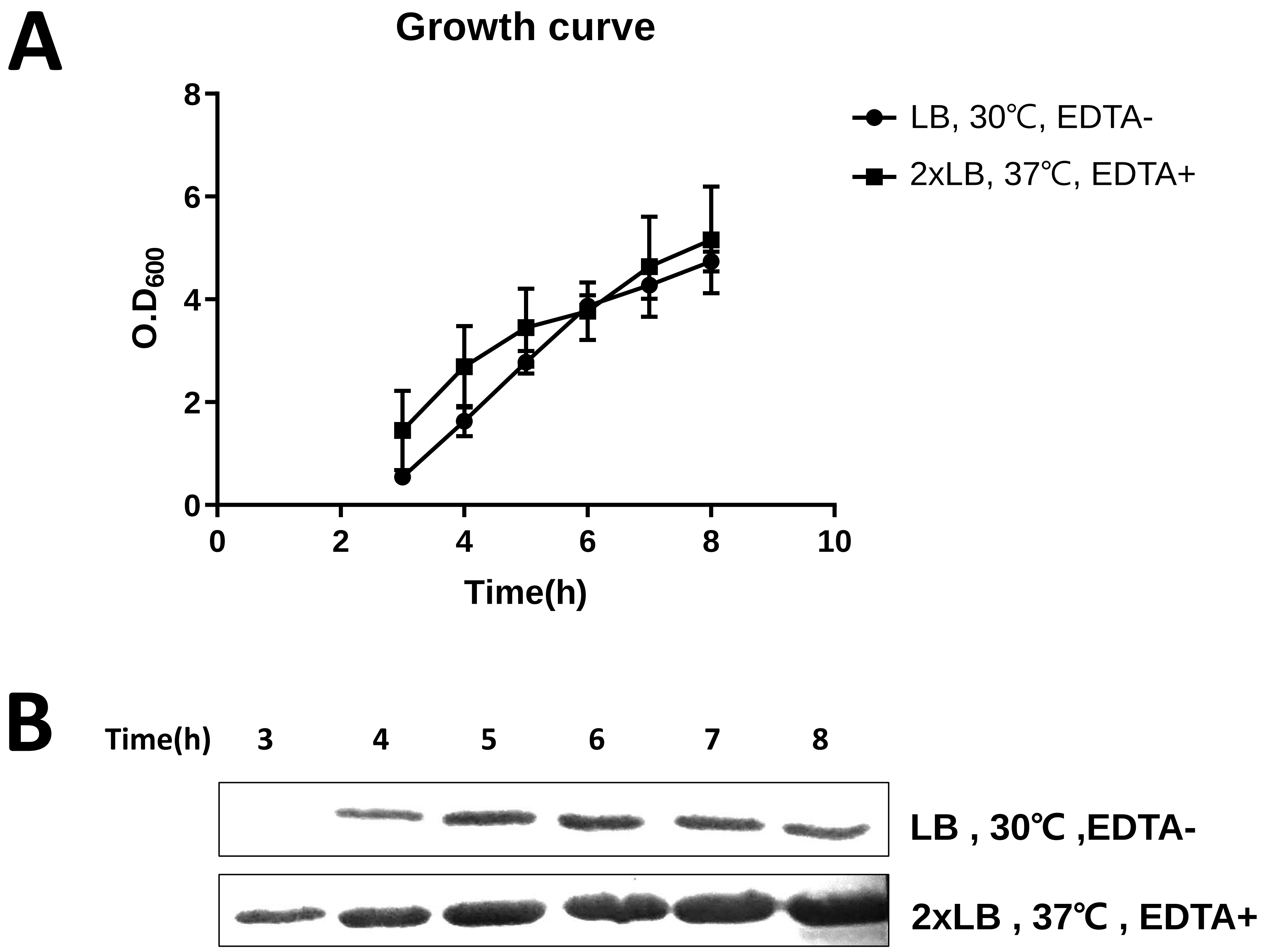

Supplementary Fig. S12. Effects of the optimized conditions on the growth and BEV production of *V. vulnificus*.

(A) Effect on the growth of cells. The data are average values from three independent experiments, and error bars denote the standard deviations. **(B)** Western hybridization using antibody against OmpU.

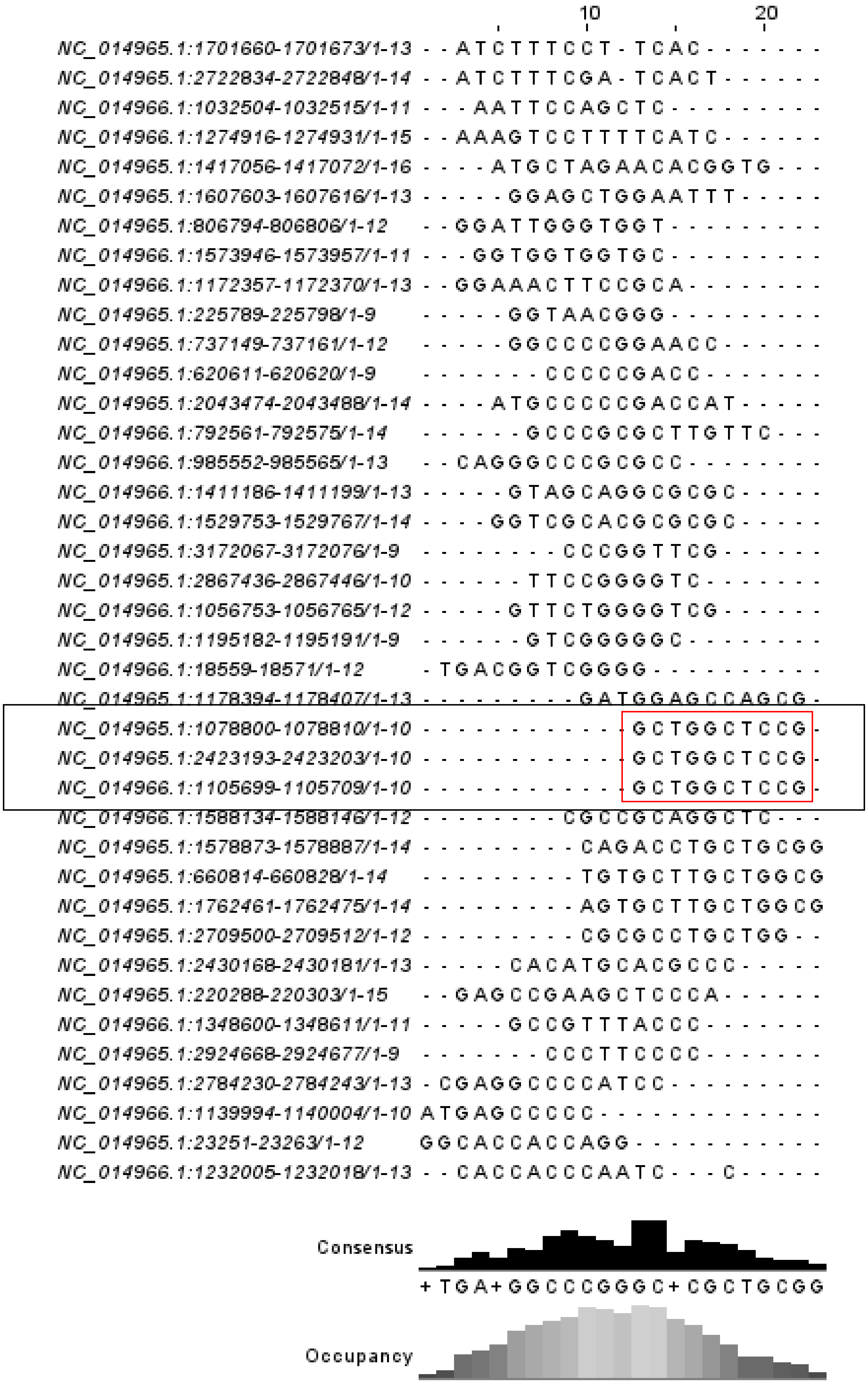

Supplementary Fig. S13. Multiple alignment of nucleotide sequences of sRNA prepared from BEVs with the 30 highest reads.

Left panel displays the accession number from the NCBI GenBank database. The next panel showed the nucleotide positions. Only aligned sequences are presented in this figure. The bottom panel provides the consensus sequences.
